# Combined Effect of Noise and Smoking on the Cognitive Performance of Automotive Industry Workers

**DOI:** 10.32598/bcn.10.5.513

**Published:** 2019-09-01

**Authors:** Iraj Alimohammadi, Fakhradin Ahmadi Kanrash, Jamileh Abolghasemi, Ali Shahbazi, Hanieh Afrazandeh, Kazem Rahmani

**Affiliations:** 1. Department of Occupational Health Engineering, School of Public Health, Iran University of Medical Sciences, Tehran, Iran.; 2. Department of Biostatistics, School of Public Health, Iran University of Medical Sciences, Tehran, Iran.; 3. Department of Neuroscience, Faculty of Advanced Technologies in Medicine, Iran University of Medical Sciences, Tehran, Iran.; 4. Department of Internal Medicine, School of Medicine, Mashhad University of Medical Sciences, Mashhad, Iran.; 5. Department of Epidemiology and Biostatistics, School of Public Health, Iran University of Medical Sciences, Tehran, Iran.

**Keywords:** Stroop test, Smoking, Noise, Occupational, Cognitive science

## Abstract

**Introduction:**

Noise is an environmental stressor and can cause or exacerbate mental disorders, and affect the individual performance in certain conditions. This study aimed to evaluate the combined effects of noise and smoking on the cognitive performance of the workers in the automotive industry.

**Methods:**

This research is a descriptive-analytical study with a cross-sectional design conducted on 300 workers randomly assigned into two groups of noise-exposed and nonexposed. They were examined using computerized tests, including the Tower of London test (TOL), Continuous Performance test (CPT), and Stroop test. The sound pressure levels were measured based on an 8-hour equal-loudness contour in each group according to ISO 9612 standard, using the Testo CEL-815 sound level meter.

**Results:**

The study of combined effects of noise and smoking on 12 CPT indicators using the 2-way Analysis of Variance (ANOVA) indicate that noise and smoking factors had a significant impact on the mean number of errors and correct responses in the third 50-stimuli stage, the mean number of errors and correct responses in the second 50-stimuli stage with P<0.001, P<0.001, P=0.012 and P<0.001 for smoking respectively, but only noise affected the other 7 indicators (P<0.001).

**Conclusion:**

Smoking and noise have negative impacts on concentration, attention, and cognitive processing speed, which can lead to an individual’s mistakes and delayed decision making at the workplace.

## Highlights

- Brain function, cognitive processing speed, and individual performance are significantly lower in smokers compared to non-smokers.
- Noise and smoking have significant effects on cognitive performance indicators.
- High noise exposure is associated with the risk of an increased number of errors in responding to the test stimuli.
- Addiction to cigarettes and other nicotine-containing products harms cognitive performance, including executive functions.

## Plain Language Summary

Excessive noise in industrial environments can cause hearing impairment, speech problems, sleep disorders, noise annoyance, and decreased efficiency of the workers. This study aimed to evaluate the combined effects of noise and smoking on the cognitive performance and psychological flexibility of workers in the automotive industry in Iran. The workers were assessed by proper tools to examine their mental performance and responses to low- or high-frequency noises. We found that long-time exposure to noise significantly affects the individual’s performance and psychomotor speed, which resulted in impaired concentration, poor working performance, and increased mistakes at work. This study revealed the relationship between smoking and brain functions in terms of response type and decision-making.

## 1. Introduction

Noise is one of the most important sources of stress in life. Loud noise may not only cause physical problems such as hearing loss and increased vulnerability, but also lowers cognitive performance (Alimohammadi, Sandrock, & Gohari, 2013; Haines, Stansfeld, Job, Berglund, & Head, 2001; Saremi et al., 2008). Noise is also known as one of the risk factors threatening human health, for example, studies have shown that noise in industrial environments can cause hearing impairment, speech problems, sleep disorders, noise annoyance, and decreased efficiency of individuals (Smith, Kane, & Popper, 2004; Zaharna & Guilleminault, 2010; Zare et al., 2009). Adverse effects of exposure to disturbing noises not only affect hearing quality but also harms mental functions that can negatively affect people’s living and working conditions (Alimohammadi & Ebrahimi, 2017).

Because noise is an environmental stressor and combined with other stressors can cause or exacerbate mental disorders and consequently affects individual’s performance in certain circumstances (Mendl, 1999), the World Health Organization (WHO) considered incidents as one of the indicators of noise-induced performance deficits. The WHO also regards environmental noise as a direct cause of mental disorders and assumes that noise exacerbates such ailments as well (Lee, Jerrett, Ross, Coogan, & Seto, 2014). Noise can directly affect an individual through synaptic nervous interactions and influence his or her emotion, cognition, and perception. In other words, exposure to noise can disrupt homeostasis and mental health (Münzel, Gori, Babisch, & Basner, 2014).

Sound has different effects on cognitive performances. Research evidence suggests that long-term exposure to noise can affect cognitive performance in central processing and language comprehension (Alimohammadi, Nassiri, Azkhosh, & Hoseini, 2010). Besides, it can harm continuous and visual attention (Haines, Stansfeld, Job, & Berglund, 1998; Sanz, García, & García, 1993). Workers exposed to long-time environmental noises have the lower hearing ability, speech ability, and memory (Hygge, Evans, & Bullinger, 1996). Studies have also shown that individual variability affects people’s efficiency, and emotional characteristics of individuals are associated with the effect of noise intensity on task performance (Bunce, MacDonald, & Hultsch, 2004).

Available studies on noise effects suggest that noise can cause occupational problems and increase the number of mistakes during working, depending on the type of sound and job. Also, meaningful and relevant information that attracts one’s attention is more likely to decrease efficiency (Bunce et al., 2004). Cognitive performance and executive functions play a fundamental role in skills and planning activities, number of errors, working memory, emotional control, concentration, inhibition, transmission, initiation, and follow-up (Cui, Wu, & She, 2009; Farhadian, Akbarfahimi, Abharian, Hosseini, & Shokri, 2017; Haines et al., 2001). As executive functions are mainly managed by the frontal lobe of the brain, these findings support the hypothesis that states “smokers may have defects in their cognitive-executive functions” (Elwood et al., 1999). In this regard, the studies conducted on executive functions and cognitive performances of substance abusers, especially cigarette smokers, showed that nicotine-containing products such as cigarettes can disrupt executive tasks at workplace, where absolute concentration is required (Kalmijn, Van Boxtel, Verschuren, Jolles, & Launer, 2002; Mortazavi et al., 2010).

Some studies on the effects of noise with different bandwidths on cognitive performance have shown that cognitive impairment is more common in people when they are exposed to the high-intensity than to the moderate-intensity sounds (Smith & Broadbent, 1985). Consequently, studying the factors that may lead to cognitive impairment is of great importance (Wright, Peters, Ettinger, Kuipers, & Kumari, 2016). Few studies have been carried out on the role and impact of using nicotine-containing products on mental health and sustained attention at workplace. This study aimed to investigate the combined effects of noise and smoking on the cognitive performance of automotive industry workers by using computer-based tests such as Tower of London (TOL), Continuous Performance Test (CPT), and Stroop test.

## 2. Methods

This research is a descriptive-analytical study with a cross-sectional design. 300 workers of the automotive industry in Tehran, Iran were randomly selected and assigned into two groups of noise-exposed (workers under exposure to a noise level of 80–86 dBA; n=150) and nonexposed (workers under exposure to ambient background noise level of 40–50 dBA; n=150). The demographic information of each group was recorded and checked if they were cigarette smokers. They were considered smokers if they had smoked 5 or more cigarettes a day for 14 years (Wild, Brewster, & Banerjee, 2005). The sound pressure levels in each group were measured based on an 8-hour equal-loudness contour (formula 1), according to ISO 9612 standard (2009).

We used the Testo CEL 815 sound level meter with a precision of 0.5 dB and ability to measure the sound intensity in the A network calibrated by Testo IEC 942/90 Class2 calibrator with an intensity of 114 dB at the frequency of 1 kHz. The subjects who had willingness to continue participation in the study were subjected to psychological and intellectual tests. Their sustained attention was measured using CPT, while for selective attention and cognitive flexibility assessment, the Stroop test was used. Also, their problem-solving ability was assessed using the TOL test. One of the inclusion criteria for entering this study was lacking underlying diseases (heart, pulmonary, and renal diseases) or metabolic and psychiatric disorders. The subjects were allowed to enter the study based on their medical records and demographic information.

1. $${\text{L}}_{\text{Epd}}(\text{dB)}=\text{10log}\left[\frac{\text{1}}{\text{8}}\sum_{1}^{1}{\text{t}}_{\text{i}}10^{\text{SPL}/10}\right]$$

### 2.1. Continuous Performance test

CPT is one of the simplest psychological tests (Halperin, Sharma, Greenblatt, & Schwartz, 1991). In all forms of this test, subjects must focus on a relatively simple visual or auditory stimulus over a period of time (only a visual stimulus was provided in this study) and present their responses by pressing a button whenever the target stimulus appeared. The goal is that the subjects use their maximum ability and perform the best performance as fast as possible. In this test, there are 150 numbers or images as stimuli, of which 20% are the target stimuli and the rest (80%) the non-target ones. The stimulus was presented for 200 ms, and the interval between the two stimuli was 1 s. The test was conducted after recording the demographic information of participants. Before the test, a training trial was carried out. At the beginning of the training trial, the examiner presented necessary explanations on the screen and fully described them. The duration of the test, including the training trial, was 200 s. For measuring the sustained attention of the participants, their correct responses, errors, omission errors, reaction time, and response time interference were recorded (Warm, 1984; Zarghi & Zarindast, 2011). The validity of this test ranges from 0.8 to 0.91 (Lezak, Howieson, Loring, & Fischer, 2004).

### 2.2. Stroop test

**Figure 1.**
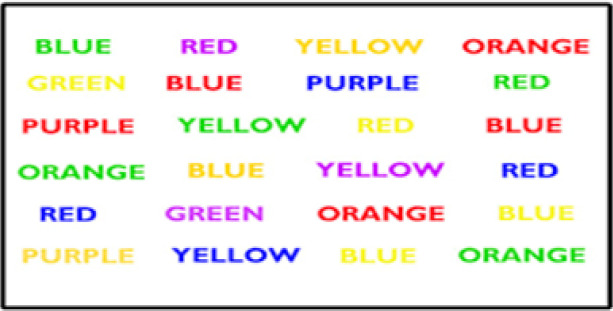
Stroop test

The Stroop test evaluates the selective attention and cognitive flexibility in various clinical groups (Stroop, 1992). The test includes two stages. First, the subject is asked to name the color of the word (written in red, blue, yellow, and green). This stage trains to identify the colors and placement of keys on the keyboard and does not affect the final result. At the second stage, a total of 96 congruent and incongruent color words are shown randomly and sequentially (Figure 1). This test measures mental flexibility, interference, and response inhibition (Wecker, Kramer, Wisniewski, Delis, & Kaplan, 2000). The interference rate is obtained by subtracting the score of correct responses to incongruent words from that of the congruent ones. Various studies have reported the validity of 0.83 for this test (Lange et al., 1992).

### 2.3. Tower of London test

**Figure 2.**
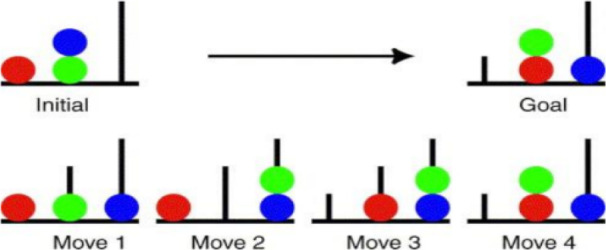
Tower of London test

The TOL test evaluates at least two executive functionings of planning and problem-solving. It is used to assess executive functions and cognitive performance of various people, including the patients with glioma, schizophrenia, and so on (Lange et al., 1992; Sturm, Fernell, & Gillberg, 2004). During the test, a picture was completed by moving the colored pages (green, blue, and red) to their right locations with a minimum number of moves. It should be noted that only the part B pages could be moved. There were three colored pages in the long column, two in the medium column, and one in the short column. The subject was allowed to solve the problem in three attempts and with a minimum number of moves according to the instructions. After success in each attempt (or if the problem was still unsolved after three attempts), the subject was asked to solve the next puzzle (Figure 2). The measured variables in this test were test duration, time delay, total time, result, error, response time, and response interference. The reliability of this test has been reported 0.79 in various studies (Lezak et al., 2004).

### 2.4. Data analysis

For analyzing the collected data, descriptive statistics (frequency, mean, and standard deviation) were used as well as the Kolmogorov-Smirnov test whose results showed the normality of the quantitative data distribution (P>0.05). tow-way Analysis of Variance (ANOVA) examined the combined effects of noise and smoking. The data were analyzed in SPSS V. 22 by considering the significance level of P<0.05.

## 3. Results

Table 1 presents the demographic characteristics of the participants. Results reported that the Mean±SD age of the subjects was (36.08±3.64) years in the noise-exposed group and (36.19±3.71) years for the nonexposed group. There was no significant difference between groups in terms of age (P=0.789). The mean duration of work experiences in nonexposed and noise-exposed groups were 14.94 and 14.99 years, respectively but this difference was not significant (P<0.84). In terms of educational level, most of the subjects in the two groups had a high school diploma (n=113, 75%), and there was a significant relationship between the two groups in terms of their educational level. Regarding marital status, the majority of the subjects were married (n=140, 99%) and there was no significant relationship between the two groups in terms of marital status (Table 1). The mean and standard deviation of TOL, Stroop, and CPT scores are presented in tables 2 to 4.

**Table 1.** Comparing the characteristics of workers in terms of exposure to noise and smoking

**Variable**		**Mean±SD**					

		**Exposed**			**Nonexposed**		

		**Smokers**	**Non-Smokers**	**Total**	**Smokers**	**Non-Smokers**	**Total**
Age (y)		35.80±3.37	36.30±3.85	36.08±3.64	36.61±3.93	36.02±3.63	36.19±3.71
Work experience (y)		14.85±1.92	15.10±2.12	14.99±2.03	15.05±1.92	14.90±2.12	14.94±2.06
Educational level		No. (%)					
High school diploma		49 (74)	64 (76)	113 (75)	6 (14)	16 (15)	22 (15)
Associate degree		16 (24)	15 (18)	31 (21)	21 (47)	39 (37)	60 (40)
Bachelor’s degree and higher		1 (0.02)	5 (0.06)	6 (0.4)	17 (39)	51 (48)	68 (45)
Marital status	Single	5 (0.7)	5 (0.6)	10 (1)	7 (16)	10 (1)	17 (11)
	Married	61 (99.3)	79 (94)	140 (99)	37 (84)	96 (90)	133 (89)

**Table 2.** Mean±SD of TOL test variables in terms of exposure to noise and smoking in samples

**Cognitive Performance Indicators**	**Mean±SD**					

	**Exposed**			**Nonexposed**		

	**Smokers**	**Non-Smokers**	**Total**	**Smokers**	**Non-Smokers**	**Total**
Test time (s)	181.29±99.281	160.92±94.92	169.88±97.06	62.86±13.45	61.66±15.06	62.01±14.57
Test delay (s)	69.80±33.58	67.61±30.21	68.57±31.64	30.14±11.52	29.90±13.48	29.97±12.90
Total time (s)	251.09±120.27	228.52±103.81	238.45±111.53	93.00±24.87	91.56±28.42	91.98±27.35
Number of errors	6.39±2.57	6.26±2.68	6.32±2.62	3.52±1.63	3.63±2.07	3.60±1.94
Test score	27.77±3.62	26.51±3.09	27.07±3.38	32.59±2.02	31.92±2.00	32.12±2.03

**Table 3.** Mean±SD of the Stroop test variables in terms of exposure to noise and smoking in samples

**Cognitive Performance Indicators**		**Mean±SD**					

		**Exposed**			**Nonexposed**		

		**Smokers**	**Non-Smokers**	**Total**	**Smokers**	**Non-Smoking**	**Total**
Congruent Stroop	Test duration (s)	56.80±7.91	58.00±7.20	57.47±7.52	44.20±8.02	44.71±8..30	44.56±8.19
	Number of errors	6.65±2.33	7.57±2.15	7.17±2.27	5.07±2.19	5.65±2.43	5.48±2.37
	Number of un-responded items	1.41±0.49	1.50±0.5	1.46±0.5	0.41±0.4	0.48±0.5	0.46±0.5
	Number of correct responses	39.94±2.40	38.93±2.26	39.37±2.37	42.52±2.28	41.87±2.38	42.06±2.36
	Response time (ms)	1238.11±74.53	1234.42±76.31	1236.04±75.30	882.30±51.14	873.21±15.24	875.87±30.59
Incongruent Stroop	Test duration (s)	66.19±5.53	66.70±5.41	66.46±5.45	53.05±5.45	51.04±5.21	51.63±5.34
	Number of errors	9.42±3.29	9.38±2.46	9.40±2.85	5.93±2.11	6.07±2.27	6.03±2.22
	Number of un-responded items	2.52±0.5	2.49±0.5	2.50±0.5	1.52±0.5	1.49±0.5	1.50±0.5
	Number of correct responses	36.06±3.32	36.13±2.42	36.10±2.88	40.55±2.36	40.44±2.43	40.47±2.41
	Response time (ms)	1428.02±57.46	1417.50±73.01	1422.13±66.61	935.34±56.53	924.74±30.14	927.85±39.82
	Interference score	4.39±2.39	3.60±2.25	3.95±2.34	2.32±1.37	2.47±1.69	2.43±1.60
	Interference time (s)	189.91±94.25	183.08±96.06	186.09±95.01	53.05±14.39	51.53±14.92	51.97±14.74

**Table 4.** Mean±SD of CPT variables in terms of exposure to noise and smoking in samples

**Cognitive Performance Indicators**		**Mean±SD**					

		**Exposed**			**Nonexposed**		

		**Smokers**	**Non-Smokers**	**Total**	**Smokers**	**Non-Smokers**	**Total**
The first 50-stimuli set	Number of errors	3.52±1.57	3.42±1.63	3.46±1.60	2.75±1.95	2.75±1.73	2.75±1.79
	Number of un-responded items	4.61±2.27	5.08±2.00	4.87±2.13	2.45±2.21	2.15±1.73	2.24±1.88
	Number of correct responses	41.73±3.27	41.38±3.15	41.53±3.20	44.07±3.48	44.68±3.15	44.50±3.25
	Response time (ms)	435.11±68.61	436.89±64.77	436.11±66.27	388.52±55.13	399.26±34.70	396.11±61.83
The second 50-stimuli set	Number of errors	8.79±1.25	7.64±1.75	8.15±1.65	3.93±2.69	2.14±1.69	2.67±2.18
	Number of un-responded items	1.64±0.85	1.60±0.93	1.61±0.89	0.82±1.1	0.59±0.54	0.66±0.75
	Number of correct responses	39.58±1.26	40.76±1.93	40.24±1.77	45.27±3.02	47.26±1.88	46.68±2.44
	Response time (ms)	542.00±27.93	531.19±53.15	535.95±44.08	428.68±35.66	427.82±25.11	428.07±28.49
The third 50-stimuli set	Number of errors	3.91±1.54	3.10±1.55	3.45±1.59	3.80±2.44	2.18±1.44	2.65±1.93
	Number of un-responded items	4.73±2.40	4.90±1.96	4.83±2.16	2.20±2.56	1.20±1.73	1.49±2.05
	Number of correct responses	41.06±3.33	42.00±3.06	41.59±3.21	44.02±3.8	46.53±2.88	45.79±3.37
	Response time (ms)	549.15±50.08	529.38±82.45	538.08±70.55	425.05±41.39	428.08±37.60	427.19±38.63

The tow-way ANOVA was used to determine the effect of noise and smoking on the cognitive performance variables. The results showed that the combined effect of noise and smoking on the cognitive performance variables was not significant (P>0.345) (Table 2). Regarding their effect on the TOL test variables, ANOVA results showed that smoking had no significant impact on these variables (P>0.09), but noise significantly affected all 5 variables in the TOL test (P<0.001) (Table 2). Regarding their effect on 12 variables in the Stroop test, ANOVA results showed that both noise (P<0.001) and smoking (P=0.008) significantly affected the mean number of errors (for congruent words) but only noise affected the other 11 variables (P<0.001) (Table 3).

The study of the combined effects of noise and smoking on 12 CPT variables using the 2-way, ANOVA indicated that both sound and smoking factors had significant effects on the number of errors and correct responses (P<0.001).

## 4. Discussion

The results of this study showed the combined effects of noise and smoking on the cognitive performance of workers in the automotive industry. The results of this study not only confirmed the impact of noise on cognitive performance but also emphasized the effect of noise on responsiveness and individual performance speed, which was considerably reduced in exposure to high levels of noise. This finding is in line with the results of the previous studies (Alimohammadi & Ebrahimi, 2017; Liu, Lin, Huang, & Chen, 2017). The environmental and acoustic factors remarkably control the speed of individual performance and cognitive process in decision-making influenced by psychomotor function (Staal, 2004). Various studies on cognitive performance also reported different results. For example, (Alimohammadi et al., 2013) reported no significant relationship between exposure to low-frequency sound (50–70 dBA) and mental performance and concluded that the extraversion and introversion personality traits could increase accuracy and decrease the duration of mental performance. The results of other studies indicated the effect of high- and low-frequency sounds on the cognitive performance of people at the workplace (Alimohammadi et al., 2013; Liu et al., 2017; Waye et al., 2002).

Regarding the effects of smoking on the cognitive performance of the workers in the automotive industry, the results of our study demonstrated the relationship between smoking and cognitive performance. Although brain function and mental performance in the work environment are affected by environmental factors such as light, sound, and temperature, some studies also investigated the effect of using nicotine-containing products such as cigarettes on the mental and brain functions in different age groups and genders and reported various results (Ernst et al., 2001; Kalmijn et al., 2002). Smoking cigarettes and other nicotine-containing products are associated with reduced mental performance as demonstrated by the CPT method in this study, and there is a strong dose-response relationship between smoking rate and mental function (Berkman et al., 1993). Smoking is a strong risk factor that can cause mental and cognitive problems such as dementia in the long term (Carmelli, Swan, Reed, Schellenberg, & Christian, 1999). Hence, avoiding these risk factors that affect people in the middle age or in various social situations that need higher brain functioning (e.g. at the workplace), can help prevent such damages in the old age (Deary et al., 2003).

The concentration which can affect an individual’s reaction time was one of the factors examined in this study in smokers and non-smokers. Smoking interfered with concentration through physiological responses to the nicotine substance in the body and significantly increased the reaction time and the correct response time in Stroop and CPT-tests. In responding to the congruent stimuli in the Stroop test, smokers showed weaker concentration compared with their responses to the incongruent stimuli, implying that concentration affects the time of response to the two different stimuli patterns.

Brain function and mental performance are affected by psychomotor speed and mental flexibility following damage or injury in the cerebral cortex capillaries; therefore, cigarette smoking can cause some changes in the vascular mechanism for blood flow and some physiological changes in the organs like brain (Mortazavi et al., 2010; Newhouse, Potter, & Singh, 2004). Thus, the smoking mechanism in patients with stroke and dementia has been well-defined through the effect of smoking on the vascular system (Cees De Groot et al., 2000; Raininko & Tilvis, 1993; Rogers, Meyer, Judd, & Mortel, 1985; Terborg, Bramer, Weiller, & Röther, 2002).

According to the study of (Kalmijn et al., 2002) on the effects of cigarette smoking on cognitive performance, the psychomotor speed and cognitive flexibility were significantly lower in smokers compared with non-smokers, indicating the physiological effects of nicotine on the cortical vascular system. This finding is consistent with our study finding regarding the reduced speed of cognitive functioning. Considering this vascular cognitive mechanism, the smokers in our study had different cognitive flexibility and psychomotor speed due to the effect of nicotine, which can interfere with concentration and result in poor working performance, as well as increased occupational errors and lack of timely decision-making. In this study, the number of correct responses and the response time to the test questions pointed out that psychomotor speed in smokers was reduced due to exposure to nicotine.

Altogether, cigarette smoking has a direct impact on workers’ cognitive functioning and reduce their mental and cognitive performance. Smokers also suffered more from concentration deficits and poor working performance compared with non-smokers. According to the results of the Stroop test and CPT-test, smokers had low-speed cognitive processing in decision making and weakness in making timely decisions.

The diagnostic skill model is one of the main reasons why concentration is more affected than attention (Alimohammadi & Ebrahimi, 2017). According to this model, attention is a less sensitive skill. This study also examined the effect of concentration on the reaction time of groups with and without exposure to noise. Exposure to noise could significantly increase the reaction and correct response time during Stroop, TOL, and CPT-tests by interfering with concentration. In the Stroop test, exposure to noise reduced concentration in responding to the incongruent stimuli more compared to the responses to congruent stimuli, implying the effect of concentration on the time of responding to the two different stimuli patterns.

The rate of human errors in high-risk and managerial jobs, where there is a need for proper concentration, has a relationship with exposure to noise such that with higher noise frequency, the human error rate increases (Ljungberg, Neely, & Lundström, 2004). This finding is consistent with our study, which showed that the workers’ more exposure to noise was associated with their increased number of errors. Previous studies have examined various factors related to the impact of noise on the cognitive performance of people, including noise characteristics and job complexity and difficulty (Staal, 2004). Generally, chronic exposure to noise significantly affects an individual’s performance and psychomotor speed, which results in impaired concentration, poor working performance and increased mistakes at work. In this study, the number of wrong and correct answers in the conducted tests confirmed that psychomotor function is disrupted after exposure to noise.

Based on the transactional model of stress, mental assessment of threatening events, or the ability to cope with stressors are related to the mental stress experienced by individuals (Stokes & Kite, 2001). Although the findings of this study can be affected by the subjective evaluation of individuals, cognitive assessment of stressors had a direct impact on the cognitive performance of workers such that positive assessments led to increased cognitive performance, and negative assessments led to decreased cognitive performance (Staal, 2004). When exposed to noise, the workers in this study had no full knowledge of the stressors affecting their cognitive performance and individual function. Awareness about these stressors (sources of noise pollution) can increase the positive effects of subjective function (e.g. following healthy behaviors), and they can work better in those settings with a control approach.

Experiencing stressful events that do not threaten their lives can also bring up positive emotions on them (e.g. lack of mental disorder) and increase the workers’ self-confidence at the workplace. In addition, studies have emphasized that the Stroop test and other cognitive/psychological tests are beneficial for the analysis of mental performance and responses to intervening stimuli that require attention (e.g. low- or high-frequency noises), and making it possible to examine the impacts on cognitive performance. The results of this study were partly consistent with the central nervous system arousal theory which is related to the stimulation of the central nervous system and the adaptation of human responses to stimuli. In our study, the samples’ physiological system could adapt to the noise in the long term and could even work better with the noise, but its consequences would reduce their accuracy and concentration in performing the assigned tasks (Mehri, Alimohammadi, Ebrahimi, Hajizadeh, & Roudbari, 2018).

In general, the reaction time in mental activities is influenced by noise. Various studies reported the effect of exposure to high- and low-frequency noises on cognitive performance. These reports indicate the influence of exposure to chronic noise on the mental and psychological conditions, including performance and processing speed. Several studies that have examined the relationship of human cognitive performance with other physical factors have also reported similar results (Haines et al., 1998; Losier, McGrath, & Klein, 1996; Sanz et al., 1993). Sound pressure levels above 65 dB can potentially affect the cognitive performance and other non-auditory functions, increase the risk of different human errors at work where people long exposed to loud noises, and cause cognitive impairment (delayed reaction time, poor attention, etc.).

In this study, the TOL test indicated a significant relationship between individual performance and strategic approaches to problem-solving and planning and noise exposure. Variables such as time delay and the number of errors showed a significant and robust relationship with noise exposure such that those exposed to chronic noise had more delays and errors in responding to this test. Mental and cognitive performances of the workers employed in industries are directly and significantly related to chronic exposure to noise. Controlling noise in industries and using personal protective equipment can remarkably and significantly reduce human errors and, on the other hand, increase efficiency and effectiveness to achieve organizational goals (Golmohammadi, Giahi, Aliabadi, & Darvishi, 2014; Malchaire, 2000).

One of the limitations of this study was the lack of individual matching, which can allow a more accurate evaluation of the study variables and eliminate the effect of confounders. Another limitation was the theoretical aspect of the issue. In this regard, the test stimuli were presented to the subjects on a computer monitor, which may not adequately examine the cognitive factors.

Noise exposure and smoking could affect the workers’ cognitive performance, thereby reducing their attention and concentration and increasing the risk of errors at work. Hence, taking into account the effect of noise and smoking on workers’ health, developing strategies for controlling smoking, and reducing noise pollution should be considered among the essential preventive measures.

## Ethical Considerations

### Compliance with ethical guidelines

All of the experiments were conducted according to the guidelines of the Ethics Committee of Iran University of Medical Sciences (Ethical code: IR.IUMS. REC1395.9411139003).
